# Supplementary material for: Protein Phosphorylation Alterations in Myotonic Dystrophy Type 1: A Systematic Review
Source: Int J Mol Sci. 2023 Feb 4;24(4):3091. doi: 10.3390/ijms24043091 (PMC9965115; doi:10.3390/ijms24043091)
Supplement: Supplementary file 1 [file ijms-24-03091-s001.zip › Protein phosphorylation in DM1 Systematic Review_SearchStrategy.pdf]

Supplementary Data 1

Protein Phosphorylation Alterations in Myotonic Dystrophy Type 1: A Systematic Review

Adriana Costa, Ana C. Cruz, Filipa Martins * and Sandra Rebelo *

Institute of Biomedicine (iBiMED), Department of Medical Sciences, University of Aveiro, 3810-193 Aveiro, Portugal
* Correspondence: samartins@ua.pt (F.M.); srebelo@ua.pt (S.R.)

webofQuestion:

1. What is the impact of protein phosphorylation in myotonic dystrophy type 1?

Search strategy

PICO (Population, Intervention, Comparison, Outcome) **framework**

P= ("Myotonic dystrophy type 1" OR "Congenital Myotonic dystrophy" OR "Dystrophia Myotonica 1" OR "Myotonic Dystrophy 1" OR "Steinert Syndrome" OR "Steinert's disease" OR "Steinert myotonic dystrophy" OR "Myotonic dystrophy of Steinert" OR "Steinert's congenital myotonic dystrophy")

I= not applicable

C= not applicable

O= ("protein phosphorylation" OR "Phosphorylation" OR "Protein Kinases" OR "Kinases" OR "Protein phosphatases" OR "Phosphatases" OR "Myotonic Dystrophy Protein Kinase" OR "DMPK" OR "Phosphoprotein" OR "Phosphotarget" OR "Protein subunits" OR "phosphopeptide" OR "proteomic")

Databases

PubMed; Web of knowledge

Typical Search – performed in title, abstract and keywords (MESH terms in Pubmed)

Search with MESH terms

("Myotonic Dystrophy"[Mesh]) AND ("Phosphorylation/genetics"[Mesh] OR "Phosphorylation/physiology"[Mesh] OR "Protein Kinases/genetics"[Mesh] OR "Protein Kinases/metabolism"[Mesh] OR "Protein Kinases/physiology"[Mesh] OR "Phosphotransferases/genetics"[Mesh] OR "Phosphotransferases/metabolism"[Mesh] OR "Phosphotransferases/physiology"[Mesh] OR "Phosphoprotein phosphatases/genetics"[Mesh] OR "Phosphoprotein phosphatases/metabolism"[Mesh] OR "Phosphoprotein phosphatases/physiology"[Mesh] OR "Myotonin-Protein Kinase/genetics"[Mesh] OR "Myotonin-Protein Kinase/metabolism"[Mesh] OR "Myotonin-Protein Kinase/physiology"[Mesh] OR "Myotonin-Protein Kinase/deficiency"[Mesh] OR "Protein subunits/genetics"[Mesh] OR "Protein subunits/metabolism"[Mesh] OR "Protein subunits/physiology"[Mesh])

MESH terms (Pubmed):

Myotonic Dystrophy/
Phosphorylation/genetics
Phosphorylation/physiology
Protein Kinases/genetics
Protein Kinases/metabolism
Protein Kinases/physiology
Phosphotransferases/genetics
Phosphotransferases/metabolism
Phosphotransferases/physiology
Phosphoprotein phosphatases/genetics
Phosphoprotein phosphatases/metabolism
Phosphoprotein phosphatases/physiology
Myotonin-Protein Kinase/genetics
Myotonin-Protein Kinase/metabolism
Myotonin-Protein Kinase/physiology
Myotonin-Protein Kinase/deficiency
Protein subunits/genetics
Protein subunits/metabolism
Protein subunits/physiology

Inclusion criteria

- include animal models of myotonic dystrophy type 1
- Include cellular models of myotonic dystrophy type 1
- Include samples from patients with myotonic dystrophy type 1
- English language

Exclusion criteria

- studies involving proxy versions
- qualitative studies, news, research protocols, case studies, case reports, theses, dissertations, abstracts, letters to the editor, unpublished work, editorials, commentaries, books, book chapters, systematic reviews (references checked), guidelines, statements and position papers
