# Supplementary material for: Protein Phosphorylation Alterations in Myotonic Dystrophy Type 1: A Systematic Review
Source: Int J Mol Sci. 2023 Feb 4;24(4):3091. doi: 10.3390/ijms24043091 (PMC9965115; doi:10.3390/ijms24043091)
Supplement: Supplementary file 1 [file ijms-24-03091-s001.zip › Supplementary Figures.pdf]

Supplementary Figures 1-5

Protein Phosphorylation Alterations in Myotonic Dystrophy Type 1: A Systematic Review

Adriana Costa [1], Ana C. Cruz [1], Filipa Martins [1] and Sandra Rebelo [1,*]

[1] Institute of Biomedicine (iBiMED), Department of Medical Sciences, University of Aveiro, 3810-193 Aveiro, Portugal

* Correspondence: srebelo@ua.pt; Tel.: +351-924-406-306; Fax: +351-234-372-587

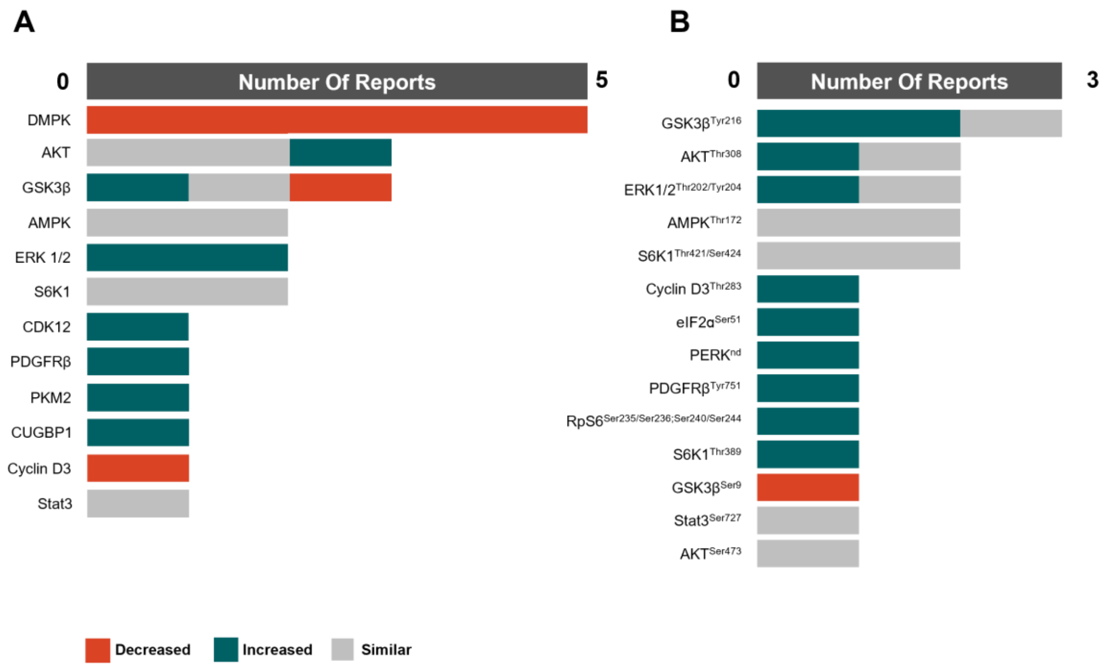

Figure S1 – Status of total and phosphorylated kinase, phosphatase and phosphoprotein levels in DM1 in human skeletal muscle tissue biopsy. Values are represented as the number of times in which a protein presented increased, similar or decreased values in DM1 in comparison with the control levels reported in the articles. A) Total protein levels in human skeletal muscle tissue biopsy; B) Phosphorylated levels in human skeletal muscle tissue biopsy; nd- not defined

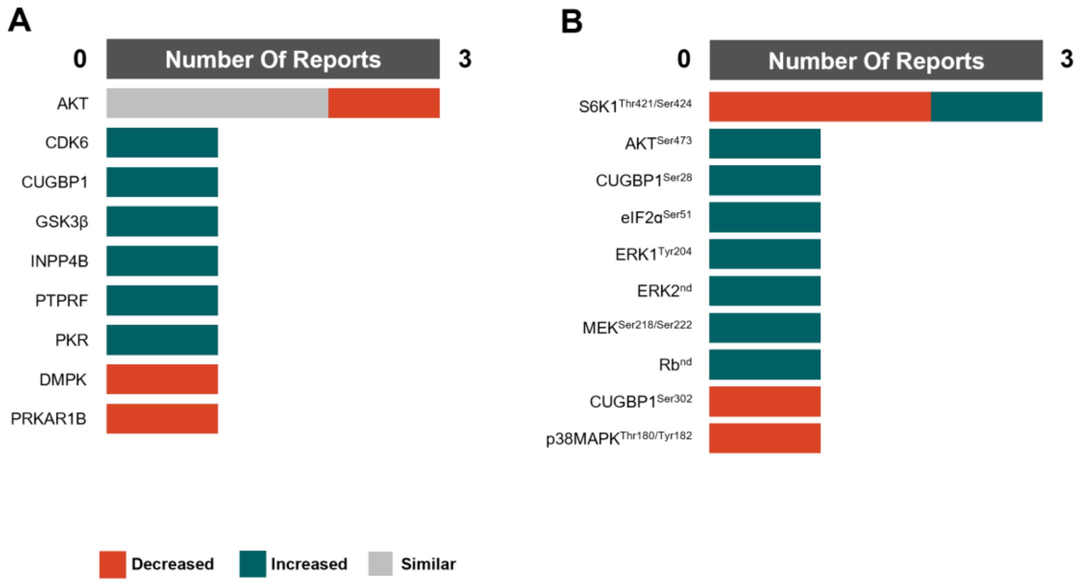

Figure S2 – Status of total and phosphorylated kinase, phosphatase and phosphoprotein levels in DM1 in human myoblasts. Values are represented as the number of times in which a protein presented increased, similar or decreased values in DM1 in comparison with the control levels reported in the articles. A) Total protein levels in human myoblasts; B) Phosphorylated levels in human myoblasts; nd- not defined

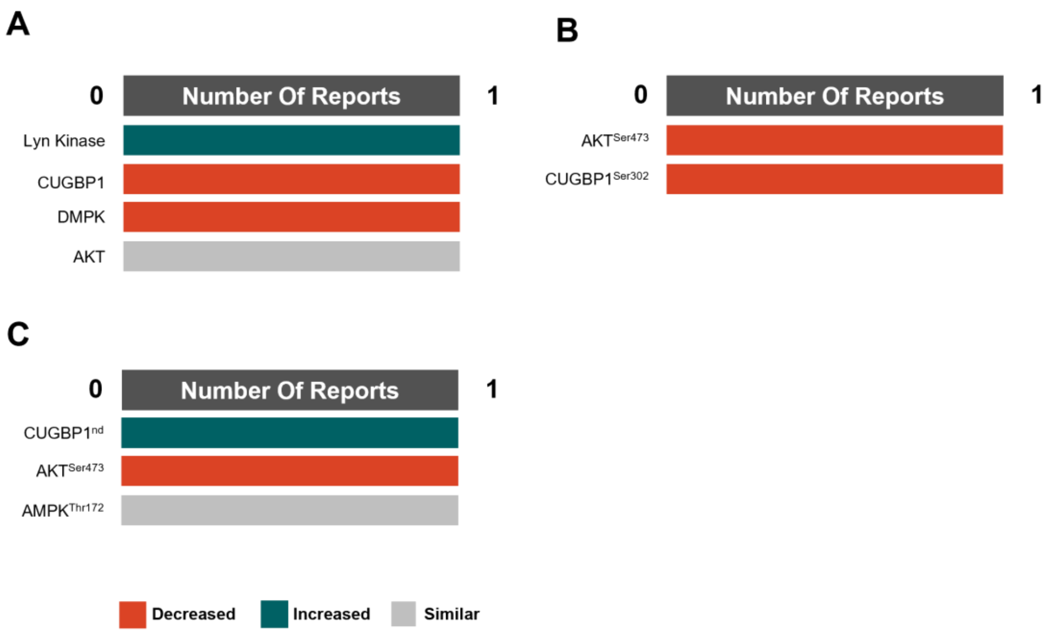

Figure S3 – Status of total and phosphorylated kinase, phosphatase and phosphoprotein levels in DM1 in human myotubes and human fibroblast-derived myotubes. Values are represented as the number of times in which a protein presented increased, similar or decreased values in DM1 in comparison with the control levels reported in the articles. A) Total protein levels in human myotubes; B) Phosphorylated levels in human myotubes; C) Phosphorylated levels in human Fibroblast-derived myotubes. nd- not defined

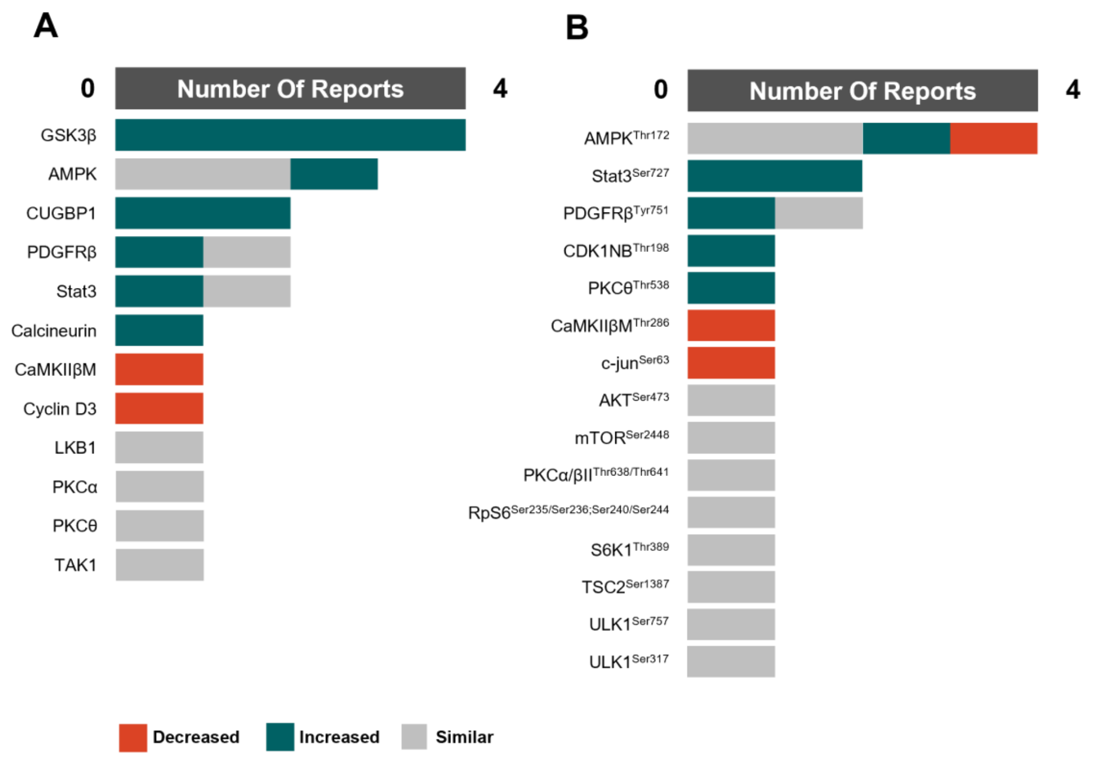

Figure S4 – Status of total and phosphorylated kinase, phosphatase and phosphoprotein levels in DM1 in mice skeletal muscle tissue biopsy. Values are represented as the number of times in which a protein presented increased, similar or decreased values in DM1 in comparison with the control levels reported in the articles. A) Total protein levels in mice skeletal muscle tissue biopsy; B) Phosphorylated levels in mice skeletal muscle tissue biopsy.

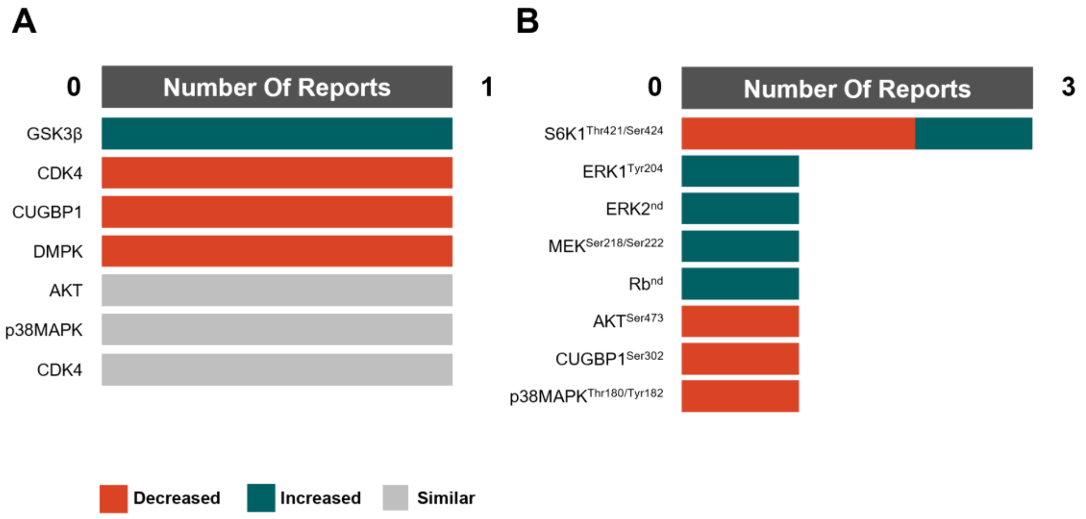

Figure S5 – Status of total and phosphorylated kinase, phosphatase and phosphoprotein levels in DM1 upon skeletal muscle differentiation. Values are represented as the number of times in which a protein presented increased, similar or decreased values in DM1 in comparison with the control levels reported in the articles. A) Total protein levels upon skeletal muscle differentiation; B) Phosphorylated levels upon skeletal muscle differentiation; nd- not defined
