# Supplementary material for: Protein Phosphorylation Alterations in Myotonic Dystrophy Type 1: A Systematic Review
Source: Int J Mol Sci. 2023 Feb 4;24(4):3091. doi: 10.3390/ijms24043091 (PMC9965115; doi:10.3390/ijms24043091)
Supplement: Supplementary file 1 [file ijms-24-03091-s001.zip › Supplementary Table_Revised Costa el al FINAL.pdf]

Supplementary Table

Protein Phosphorylation Alterations in Myotonic Dystrophy Type 1: A Systematic Review

Adriana Costa, Ana C. Cruz, Filipa Martins * and Sandra Rebelo *

Institute of Biomedicine (iBiMED), Department of Medical Sciences, University of Aveiro,
3810-193 Aveiro, Portugal
* Correspondence: samartins@ua.pt (F.M.); srebelo@ua.pt (S.R.)

Table S1. Summary data of the included studies of the systematic review.

Author, Year, Country	*Sample type and characterization*	*Evaluation method*	*Phosphorylation associated proteins*	*Main findings*
**André et al. 2019, The Netherlands** [23]	DM1: Human congenital DM1 proliferating myoblasts; CTG repeat length: 2600<br>CTL: Human congenital DM1 proliferating myoblasts with excision of CTG repeats	Mass spectrometry	CDK6	Total CDK6 level: Significantly **increased** in DM1 (fold change: 0.66; $p<0.05$) vs CTL.
			INPP4B	Total INPP4B level: Significantly **increased** in DM1 (fold change: 0.66; $p<0.05$) vs CTL.
			PRKAR1B	Total PRKAR1B level: Significantly **decreased** in DM1 (fold change: -0.59; $p<0.05$) vs CTL.
			PTPRF	Total PTPRF level: Significantly **increased** in DM1 (fold change: 0.64; $p<0.05$) vs CTL.
**Beffy et al. 2010, Italy** [24]	DM1: Human fetus-derived myoblasts; CTG repeat length: 3200<br>CTL: Human fetus-derived myoblasts; CTG repeat length: 20	Western blot	DMPK	Total DMPK level: **Decreased** in DM1 during all the differentiation process (d0-d6).<br>Semi-quantitative total DMPK level (fold change):<br>d0: DM1: 0.3; CTL: 0.5; d2: DM1: 0.3; CTL: 0.7; d4: DM1: 0.3; CTL: 0.6; d6: DM1: 0.3; CTL: 0.5 (p: NR)
			ERK1	p-ERK1 level: Significantly **increased** levels of p-ERK1 (Tyr204) in DM1 vs CTL from d0 to d4 of differentiation. At d6, the levels p-ERK1 (Tyr204) **decrease** to the same background level as CTL. (p: NR)
			ERK2	p-ERK2 level: **Increased** p-ERK2 in DM1 vs CTL, in d0 and d2 of differentiation.<br><br>p-ERK2/Total ERK2 ratio:<br>d0: DM1 > CTL ($p<0.01$); d2: DM1 > CTL ($p<0.01$); d4: DM1 >CTL (p=ns); d6: DM1 >CTL (p=ns)<br>DM1: d0> d2 > d4 > d6; CTL: d0 < d2 < d4 > d6
			MEK	p-MEK level: **Increased** p-MEK (Ser 218/Ser222) in DM1 vs CTL, in d0 and d2 of differentiation.

				p-MEK/Total MEK ratio:<br>d0: DM1 > CTL (p<0.01); d2: DM1 > CTL (p<0.05); d4: DM1 > CTL (p=ns); d6: DM1 > CTL (p=ns)<br>DM1: d0>d2>d4>d6; CTL: d0<d>d4~d6
			p38MAPK	Total p38MAPK level: **Similar** between DM1 vs CTL during differentiation.<br>p-p38MAPK level: **Decreased** (50%) p-p38MAPK (Thr180/Tyr182) in DM1 during differentiation.<br><br>p-p38MAPK/Total p38MAPK ratio:<br>d0: DM1 < CTL (p<0.05); d2: DM1 < CTL (p<0.05); d4: DM1 < CTL (p<0.05);<br>d6: DM1 < CTL (p<0.05)<br>DM1: d0 > d2 > d4 > d6; CTL: d0 < d2 > d4 < d6
			Rb	p-Rb level: **Increased** in p-Rb during day 0 and day 2 of differentiation in DM1 vs CTL. **In both** DM1 and CTL differentiation, Rb protein is completely **dephosphorylated** at day 4 and 6 upon incubation with differentiating medium.<br><br>p-Rb/dp-Rb ratio (fold change):<br>d0: DM1: 0.78 vs CTL: 0.34 (p:NR); d2: DM1: 0.56 vs CTL: 0.27 (p:NR);<br>d4: DM1: n.d vs CTL: 0.15 (p:NR); d6: DM1: n.d vs CTL: n.d (p:NR)
			S6K1	p-S6K1 level: Significantly **reduced** levels of p-S6K1 (Thr421/Ser424) during day 0,2, and 4 of differentiation in DM1 vs CTL.<br><br>p-S6K1/Total S6K1 ratio:<br>d0: DM1 < CTL (p<0.05); d2: DM1 < CTL (p<0.01); d4: DM1 < CTL (p<0.01); d6: DM1 < CTL (p=ns)<br>DM1: d0 < d2 ~ d4 > d6; CTL: d0 < d2 > d4 > d6
**Botta et al. 2013, Italy** [25]	DM1: Human *vastus lateralis* differentiated myotubes; n=2; CTG Repeat length (range): 800-1500 (n=8)<br><br>CTL: Human *vastus lateralis* differentiated myotubes from subjects without neuromuscular disorders; n=3	Western Blot; Immunofluorescence	Lyn kinases	Total Lyn level: Significantly **increased** Lyn protein levels and nuclear localization (570%) in DM1 vs CTL.<br><br>Lyn activity: Significantly **increased** nuclear activity and Tyr phosphorylation activity (250%) in DM1 vs CTL.<br><br>Semi-quantitative total Lyn level: DM1 > CTL (p<0.02)<br>Semi-quantitative p-Tyr phosphorylation: DM1 > CTL (p<0.02)
**Brockhoff et al. 2017, Switzerland** [26]	DM1: HSA^{LR} mice skeletal muscle tissue biopsies; Age: 2-month-old; CTG repeat Length: 250<br>CTL: Background strain (FVB/N) mice skeletal muscle tissue biopsies	Western blot	AKT	p-AKT level: **Similar** p-AKT (Ser473) levels between fed and 24h starvation mice in DM1 vs CTL.<br><br>p-AKT/Total AKT ratio:<br>Fed: DM1 ~ CTL (p=ns); 24hStarvation: DM1 > CTL (p=ns)

	Fed Conditions - n= 3<br>24h Starvation Conditions- n= 4			Fed DM1 > 24hStarvation DM1 (p<0.05); Fed CTL > 24hStarvation CTL (p<0.05)
			CaMKIIβM	Total CaMKIIβM level: **Decreased** levels of CaMKIIβM in DM1 vs CTL. (p: NR)<br><br>p-CaMKIIβM level: **Decreased** levels of p-CaMKIIβM (Thr286) in DM1 vs CTL in fed and 24h starvation mice.<br><br>p-CaMKIIβM fold change:<br>Fed: DM1 < CTL (p<0.05); 24hStarvation: DM1 < CTL (p<0.05)<br>Fed DM1 ~ 24hStarvation DM1 (p=ns); Fed CTL ~ 24hStarvation CTL (p=ns)
	DM1: HSA^{LR} mice skeletal muscle tissue biopsies; n=3; Age: 2-month-old; CTG repeat length: 250<br>CTL: FVB/N mice skeletal muscle tissue biopsies; n=4		AMPK	p-AMPK level: **Similar** p-AMPK (Thr172) levels between fed and 45h starvation DM1 vs CTL. **Reduced** levels of p-AMPK (Thr172) in 24h starvation DM1 mice vs CTL.<br><br>p-AMPK/Total AMPK ratio:<br>Fed: DM1 > CTL (p=ns); 24h Starvation: DM1 < CTL (p<0.05);<br>45hStarvation: DM1 < CTL (p=ns)
	DM1: HSA^{LR} mice skeletal muscle tissue biopsies; Age: 2-month-old; CTG repeat length: 250<br>CTL: FVB/N mice skeletal muscle tissue biopsies<br>Fed Conditions -n= 3<br>24hStarvation Conditions - n= 7		RpS6	p-RpS6 level: **Increased** levels of p-RpS6 (Ser235/Ser236; Ser240/Ser244) in DM1 vs CTL in 24h starvation mice. **Similar** levels in fed and 45 h starvation in DM1 vs CTL.<br><br>p-RpS6/Total RpS6 ratio:<br>Fed: DM1 > CTL (p=ns); 24h Starvation: DM1 > CTL (p<0.001);<br>45h Starvation: DM1 < CTL (p=ns)<br><br>Fed DM1 > 24hStarvation DM1 (p=ns); Fed CTL > 24hStarvation CTL (p<0.01)<br>Fed DM1 > 45hStarvation DM1 (p<0.01); Fed CTL > 45hStarvation CTL (p<0.05)
	DM1: HSA^{LR} mice skeletal muscle tissue biopsies; Age: 2-month-old; CTG repeat length: 250<br>CTL: FVB/N mice skeletal muscle tissue biopsies		LKB1	Total LKB1 level: **Similar** in DM1 vs CTL in fed and 24h starvation mice. (p: NR)
			TAK1	Total TAK1 level: **Similar** in DM1 vs CTL in fed and 24h starvation mice. (p: NR)
			mTOR	p-mTOR level: **Similar** levels of p-mTOR (Ser2448) between fed and 24h starvation mice in DM1 vs CTL.<br><br>p-mTOR/mTOR ratio:<br>Fed: DM1 > CTL (p=ns); 24h Starvation: DM1 < CTL (p=ns)
			S6K1	p-S6K1 level: **Similar** levels of p-S6K1 (Thr389) in fed DM1 vs fed CTL mice. **Increased** levels of p-S6K1 (Thr389) in 24h starvation DM1 vs CTL. (p: NR)
			TSC2	p-TSC2 level: **Similar** levels of p-TSC2 (Ser1387) in DM1 vs CTL fed and 24h starvation mice. (p: NR)

			ULK1	p-ULK1 level: **Slight but not significant increase** of p-ULK1 (Ser757) in DM1 vs CTL in 24h starvation mice (p: NR). **Similar** levels of p-ULK1 (Ser317) in DM1 vs CTL in fed and 24h starved mice. (p: NR)<br><br>p-ULK1/Total ULK1 ratio (Ser757):<br>Fed: DM1 < CTL (p=ns); 24h Starvation: DM1 > CTL (p=ns);<br><br>Fed DM1 > 24hStarvation DM1 (p=ns); Fed CTL > 24hStarvation CTL (p<0.01)<br><br>p-ULK1/Total ULK1 ratio (Ser317):<br>Fed: DM1 < CTL (p=ns); 24h Starvation: DM1 < CTL (p=ns);<br><br>Fed DM1 < 24hStarvation DM1 (p=ns); Fed CTL < 24hStarvation CTL (p=ns)
	DM1: Human patients skeletal muscle tissue biopsies; n=3<br>CTL: Human skeletal muscle tissue biopsies; n=5		AMPK	Total AMPK level: **Similar** levels in DM1 vs CTL (p: NR)<br><br>p-AMPK level: **Similar** levels of p-AMPK (Thr172) in DM1 vs CTL. (p: NR)
			AKT	Total AKT level: **Similar** levels in DM1 vs CTL. (p: NR)<br><br>p-AKT level: **Similar** levels of p-AKT (Ser473) in DM1 vs CTL. (p:NR)
			S6K1	p-S6K1 level: **Increased** levels of p-S6K1 (Thr389) in DM1 vs CTL. (p: NR)
			RpS6	p-RpS6 level: **Increased** levels of p-RpS6 (Ser235/Ser236; Ser240/Ser244) in DM1 vs CTL. (p: NR)
	DM1: Human patients fibroblast-derived myotubes; n=3<br>CTL: Human fibroblast-derived myotubes		AKT	p-AKT level: **Similar** levels of p-AKT (Ser473) in DM1 vs CTL upon starvation. (p: NR)
			AMPK	p-AMPK level: **Similar** levels of p-AMPK (Thr172) in DM1 vs CTL upon starvation. (p: NR)
			RpS6	p-RpS6 level: **Increased** levels of p-RpS6 (Ser235/Ser236; Ser240/Ser244) in DM1 vs CTL upon starvation (p: NR).
**Denis et al. 2013, France** [27]	DM1: Human neural stem cells with large CTG expansions; n=2<br><br>CTL: Human neural stem cells; n=1	Western blot	AKT	Total AKT levels: **Similar** levels between DM1 vs CTL.<br><br>p-AKT levels: **Similar** levels of p-AKT (Ser473) between DM1 vs CTL<br><br>p-AKT/Total AKT ratio: DM1 < CTL (p=ns)
			AMPK	Total AMPK levels: **Similar** levels between DM1 vs CTL.<br><br>p-AMPK levels: **Similar** levels of p-AMPK (Thr172) between DM1 vs CTL.<br><br>p-AMPK/ Total AMPK ratio: DM1 < CTL (p=ns)
			GSK3α/β	Total GSK3α/β level: **Similar** levels between DM1 vs CTL.

				p-GSK3α/β level: **Decreased** levels of p-GSK3α/β (Ser21/Ser9) between DM1 vs CTL.<br><br>p-GSK3α/ Total GSK3α ratio: DM1 < CTL ($p<0.05$)<br><br>p- GSK3β/Total GSK3β ratio: DM1 < CTL ($p<0.05$)
			Rb	p-Rb level: **Decreased** levels of p-Rb (Ser801/Ser811) in DM1 vs CON.<br><br>p-Rb/Total Rb ratio: DM1 < CTL ($p<0.05$)
			RpS6	Total RpS6 level: **Similar** levels between DM1 vs CTL.<br><br>p-RpS6 level: **Decreased** levels of p-RpS6 (Ser235/Ser236 and Ser240/Ser245) between DM1 vs CTL.<br><br>p-RpS6 (Ser235/Ser236)/Total RpS6 ratio: DM1 < CTL ($p<0.001$)<br><br>p-RpS6 (Ser240/Ser245)/Total RpS6 ratio: DM1 < CTL ($p<0.05$)
**Furling et al. 2003, France and UK** [28]	DM1: Human fetal congenital DM1 skeletal muscle tissue biopsies; n=5; CTG repeat length (range): 1800-3700<br>CTL: Human fetal skeletal muscle tissue biopsies; n=9	Western blot	DMPK	Total DMPK levels: **Decreased** levels in DM1 vs CTL.<br><br>Semi-quantitative DMPK levels (fold change): DM1: 0.89 vs CTL: 1.57 (Reduction to 57% of control; p=0.006)
	DM1: Human fetal congenital DM1 myotubes;<br>CTL: Human fetal myotubes without neuromuscular diseases		DMPK	Total DMPK levels: **Decreased** levels in DM1 vs CTL (Reduction of 53% of control values; p: NR)
**Gao et al. 2013, USA** [29]	DM1: Human DM1 skeletal muscle tissue biopsies; n=9<br>CTL: Human skeletal muscle tissue biopsies; n=7	Western blot<br>Immunohistochemistry	PKM2	Total PKM2 levels: **Increased** levels in DM1 vs CTL. **Increased** presence in type 1 myofibers. (p: NR)
	DM1: Mouse C2C12 myoblasts with CUGexp RNA<br>CTL: Mouse C2C12 myoblasts			Total PKM2 levels: **Increased** levels in DM1 vs CTL. (p: NR)
**García-Puga et al. 2020, Spain** [30]	DM1: Patient skin-derived fibroblasts; n=3; Age (Range): 34-71; CTG repeat length (Range): 167-1650 (n=7)	Western blot	AKT	p-AKT levels: **Decreased** levels of p-AKT (Ser473) in DM1 vs CTL.<br><br>Semi-quantitative p-AKT levels: DM1 < CTL (p: NR)

	<u>CTL:</u> Control skin-derived fibroblasts; n=3; Age (Range): 48-53		DMPK	<u>Total DMPK levels:</u> **Decreased** levels in DM1 vs CTL.<br><br><u>Semi-quantitative total DMPK levels:</u> DM1 < CTL ($p<0.01$)
			p38MAPK	<u>p-p38MAPK levels:</u> **Increased** levels of p-p38MAPK (Thr180/Tyr182) in 2-fold in DM1 vs CTL.<br><br><u>p-p38MAPK/Total p38MAPK ratio:</u> DM1 > CTL ($p<0.05$)
**Huichalaf et al. 2010, USA** [31]	<u>DM1:</u> Human primary *biceps brachii* myoblasts; n=1; CTG repeat length: 320<br><u>CTL:</u> Human primary *biceps brachii* myoblast cells	Western blot	CUGBP1	<u>Total CUGBP1 levels</u>: **Increased** levels in DM1 vs CTL. (p: NR)<br><br><u>p-CUGBP1 levels:</u> **Reduced** levels of p-CUGBP1 (Ser302) in DM1 vs CTL. (p: NR)
			eIF2α	<u>p-eIF2α levels:</u> **Increased** levels of p-eIF2α (Ser51) in DM1 vs CTL. (p: NR)
			PKR	<u>Total PKR levels:</u> **Increased** levels in DM1 vs CTL. (p: NR)
	<u>DM1</u>: CHO-tTA cells with 960 CUGexp RNA<br><u>CTL</u>: CHO cells without CUGexp RNA		CUGBP1	<u>Total CUGBP1 levels</u>: **Increased** levels in DM1 vs CTL. (p: NR)
			eIF2α	<u>p-eIF2α levels:</u> **Increased** levels of p-eIF2α (Ser51) in DM1 vs CTL. (p: NR)
**Ikezoe et al. 2007, Japan** [32]	<u>DM1:</u> Human patients *biceps brachii* skeletal muscle tissue biopsies; n=5; CTG repeat length (range): 100-1200	Immunohistochemistry	eIF2α	<u>p-eIF2α levels:</u> **Increased** levels of p-eIF-2α (Ser51) in 4 of 5 patients. (p: NR)
			PERK	<u>p-PERK levels:</u> **Increased** levels of p-PERK in 4 of 5 patients. (p: NR)
**Jones et al. 2012, USA** [33]	<u>DM1:</u> Human patients *biceps brachii* skeletal muscle tissue biopsies; n=8; Age (range): 42-46<br><br><u>CTL:</u> Healthy participants *biceps brachii* skeletal muscle tissue biopsies; n=5; Age (range): 46-54	Western blot	CUGBP1	<u>Total CUGBP1 levels:</u> **Increased** in DM1 vs CTL.<br><br><u>Semi-quantitative total CUGBP1 levels:</u> DM1 > CTL (p: NR)
			Cyclin D3	<u>Total Cyclin D3 levels:</u> **Decreased** in DM1 vs CTL.<br><br><u>p-Cyclin D3 levels:</u> **Increased** levels of p-Cyclin D3 (Thr283) in DM1 vs CTL.<br><br><u>Semi-quantitative total Cyclin D3 levels</u>: DM1 < CTL (p: NR)<br><u>p-Cyclin D3/Total Cyclin D3 ratio:</u> DM1 > CTL (p: NR)
			GSK3β	<u>Total GSK3β levels:</u> **Increased** in DM1 vs CTL.<br><br><u>p-GSK3β levels:</u> **Increased** levels of p-GSK3β (Tyr216) in DM1 vs CTL. **Decreased** levels of p-GSK3β (Ser9) in DM1 vs CTL.<br><br><u>Semi-quantitative total GSK3β levels</u>: DM1 > CTL (p: NR)<br><u>Semi-quantitative p-GSK3β (Ser9) levels:</u> DM1 < CTL (p: NR)

	DM1: Human DM1 myoblasts; n=8; Age (range): 42-46<br><br>CTL: Human myoblasts; n=5; Age (range): 46-54		GSK3β	Total GSK3β levels: **Increased** levels of cytoplasmic and nuclear GSK3β in DM1 vs CTL during all steps of myoblast differentiation (p: NR).
	DM1: HSA^{LR} mice skeletal muscle tissue biopsies; n=6; Age: 6-months-old<br>CTL: WT mice skeletal muscle tissue biopsies; Age: 6-months-old		CUGBP1	Total CUGBP1 levels: **Increased** levels in DM1 vs CTL.<br><br>Semi-quantitative total CUGBP1 levels: DM1 > CTL (p:NR)
			Cyclin D3	Total Cyclin D3 levels: **Decreased** levels in DM1.<br><br>Semi-quantitative total Cyclin D3 levels: DM1 < CTL (p: NR)
			GSK3β	Total GSK3β levels: **Increased** levels in DM1 vs CTL.<br><br>Semi-quantitative total GSK3β levels: DM1 > CTL (p: NR)
	DM1: HSA^{LR} mice skeletal muscle tissue biopsies; n=6; Age: 1-month-old<br>CTL: WT mice skeletal muscle tissue biopsies; Age: 1-month-old		GSK3β	Total GSK3β levels: **Increased** levels in DM1 vs CTL.<br>Semi-quantitative total GSK3β: DM1 < CTL (p: NR)
**Ketley et al. 2020, UK** [34]	DM1: Human patients *vastus lateralis* skeletal muscle tissue biopsies; n=4<br>CTL: Healthy participants *vastus lateralis* skeletal muscle tissue biopsies; n=4	Western blot	CDK12	Total CDK12 levels: **Increased** levels in DM1 vs CTL in 48%.<br>Semi-quantitative total CDK12 levels: DM1 > CTL (p=0.02)
**Khajavi et al. 2001, USA** [35]	DM1: Human patients LBCLs; n=18	Western blot	AKT	Total AKT levels: **Not significantly different** levels across different CTG repeat lengths. (p: NR)<br><br>p-AKT levels: **Not significantly different** levels of p-AKT (Ser473) in DM1 that correlated with CTG Repeat length. (p: NR)
			ERK 1/2	Total ERK1/2 levels: **Not significantly different** across different CTG repeat lengths. (p: NR)<br><br>p-ERK 1/2 levels: **Increased levels** of p-ERK 1/2 (Thr202/Tyr204) in DM1 that correlated with CTG Repeat length. (p: NR)<br><br>Semi-quantitative p-ERK1/2 levels (fold change):<br>DM1 550 repeats: 0.27 vs DM1 740 repeats: 0.42 vs DM1 750 repeats: 0.50 vs DM1 770 repeats: 0.63 vs DM1 1150 repeats: 0.95

**Kim et al. 2016, USA** [36]	DM1: DM5 mice skeletal muscle tissue biopsies with CUGexp RNA and doxycycline induction; n (range)= 3-5<br><br>CTL: DM5 mice skeletal muscle tissue biopsies with CUGexp RNA and without doxycycline induction; n (range)=3-5	Western blot	CUGBP1	Total protein levels: **Increased** levels in DM1 vs CTL.<br><br>Semi-quantitative total CUGBP1 levels: DM1 > CTL (p=0.05)
			GSK3β	Total GSK3β: **Increased** in DM1 vs CTL<br><br>Semi-quantitative total GSK3β relative levels (fold change): DM1: 2.4 vs CTL: 1 (p: NR)
			PKC	Total PKCα levels: **Similar** in DM1 vs CTL.<br><br>Total PKCθ levels: **Similar** in DM1 vs CTL.<br><br>p-PKCα/βII levels: **Similar** in p-PKCα/βII (Thr638/Thr641) in DM1 vs CTL.<br><br>p-PKCθ levels: **Increase** in p-PKCθ (Thr538) in DM1 vs CTL.<br><br>Semi-quantitative total PKCα levels: DM1 < CTL (p=ns)<br>Semi-quantitative total PKCθ levels: DM1 < CTL (p=ns)<br>Semi-quantitative p-PKCα/βII levels: DM1 < CTL (p=ns)<br>Semi-quantitative p-PKCθ levels: DM1 > CTL (p=0.05)
**Kuyumcu-Martinez et al. 2007, USA** [37]	DM1: Human patients heart tissue<br>CTL: Human heart tissue from unaffected individuals	Western blot	CUGBP1	p-CUGBP1 levels: **Increased** phosphorylation in DM1 vs CTL. (p: NR)
			PKC	p-PKC levels: 3 out of 4 DM1 patients with **activation** of p-PKCα and p-PKCβII in compared to CTL. (p: NR)
	DM1: Human patients fibroblast-derived myotubes; CTG repeat length: 2000<br>CTL: Human fibroblast-derived myotubes from unaffected individuals		CUGBP1	p-CUGBP1 levels: **Increased** rphosphorylation in DM1 vs CTL. (p: NR)
	DM1: TAM mice cardiac tissue<br>CTL: Mice cardiac tissue		CUGBP1	Total CUGBP1 levels: **Increased** steady-state levels in DM1 vs CTL. (p: NR)<br><br>p-CUGBP1 levels: **Increased** phosphorylation in DM1 vs CTL. (p: NR)
	DM1: COS M6 cells with 960 CUGexp RNA<br>CTL: COS M6 cells without CUGexp RNA	Western blot; Kinase Assay	PKC	p-PKC levels: **Increased** levels of p-PKCα and p-PKCβII in DM1 vs CTL. (p: NR)
**Llagostera et al. 2007, Spain** [38]	DM1: DMPK knockout mice cardiac tissue<br>CTL: WT mice cardiac tissue	Western blot	AKT	p-AKT levels: **Decreased** levels in 39±5% of p-AKT (Ser473) in DM1 vs CTL upon activation of the insulin pathway (p<0.05).
			GSK3β	p-GSK3β levels: **Decreased** levels in 67±9% of p-GSK3β (Ser9) in DM1 vs CTL upon activation of the insulin pathway (p<0.05).
			InsR/IGF-1R	p-Insulin/IGF-1 receptor levels: **Decreased** levels in 54±10% of p-InsR (Tyr1150/Tyr1151) and p-IGF-IR (Tyr1135/Tyr1136) in DM1 vs CTL upon activation of the insulin pathway (p<0.05).

	DM1: DMPK knockout mice skeletal muscle tissue biopsies<br>CTL: WT mice skeletal muscle tissue biopsies		InsR/IGF-1R	p-Insulin/IGF-1 receptor levels: **Decreased** levels in 44±10% of p-InsR (Tyr1150/Tyr1151) and p-IGF-IR (Tyr1135/Tyr1136) in DM1 vs CTL upon activation of the insulin pathway ($p<0.05$).
**Llagostera et al. 2012, Spain** [39]	DM1: DMPK knockout mice cardiac tissue<br>CTL: WT mice cardiac tissue	Western blot	PLN	p-PLN levels: **Increase** in 4-fold in the levels of p-PLN (Ser16) in CTL in response to isoproterenol (β-adrenegic agonist). In DMPK knockout mice, p-PLN (Ser16) levels were **unaltered**.<br><br>p-PLN/Total PLN ratio:<br>Without isoproterenol activation: DM1 < CTL ($p<0.05$)<br>With isoproterenol activation: DM1 < CTL ($p<0.05$)
**Morriss et al. 2018, USA** [40]	DM1: TREDT960I mice skeletal muscle tissue biopsies + dox; n=12<br>DM1 mice with moderate muscle wasting - n= 7<br>DM1 mice with severe muscle wasting - n= 5<br><br>CTL: TREDT960I mice skeletal muscle tissue biopsies lacking the reverse transactivator + dox; n=8<br>CTL mice with moderate muscle wasting - n= 4<br>CTL mice with severe muscle wasting -n= 4	RPPA analysis/Western blot	AMPKα	Total AMPKα levels: **Similar** levels in DM1 vs CTL mice with moderate muscle wasting. **Increased** levels of AMPKα (F6) in severe muscle wasting mice in DM1 vs CTL.<br><br>p-AMPKα levels: **Increased** levels of p-AMPKα (Thr172) in DM1 vs CTL mice with severe muscle wasting. **Similar** levels of p-AMPKα (Thr172) in DM1 vs CTL mice with moderate muscle wasting.<br><br>Semi-quantitative total AMPKα (23A3) levels:<br>Moderate muscle wasting: DM1 < CTL (p=ns)<br>Severe muscle wasting: DM1 < CTL (p=ns)<br><br>Semi-quantitative total AMPKα (F6) levels:<br>Moderate muscle wasting: DM1 < CTL (p=ns)<br>Severe muscle wasting: DM1 > CTL ($p=0.05$)<br><br>Semi-quantitative p-AMPKα levels:<br>Moderate muscle wasting: DM1 < CTL (p=ns)<br>Severe muscle wasting: DM1 < CTL ($p<0.01$)<br><br>p-AMPKα (23A3)/Total AMPKα ratio:<br>Moderate muscle wasting: DM1 > CTL (p=ns)<br>Severe muscle wasting: DM1 > CTL ($p<0.01$)<br><br>p-AMPKα (F6)/Total AMPKα ratio:<br>Moderate muscle wasting: DM1 > CTL ($p<0.01$)<br>Severe muscle wasting: DM1 > CTL (p=ns)
		RPPA	c-Jun	p-c-Jun levels: **Decreased** levels of p-c-Jun (Ser63) in DM1 vs CTL. (p: NR)

		RPPA	CDKN1B	p-CDKN1B levels: **Increased** levels of p-CDKN1B (Thr198) in DM1 vs CTL. (p: NR)
		RPPA analysis/Western blot	PDGFRβ	Total PDGFRβ levels: **Increased** levels in DM1 vs CTL mice with severe muscle wasting. **Similar** in DM1 vs CTL mice with moderate muscle wasting.<br><br>p-PDGFRβ levels: **Increased** levels of p-PDGFRβ (Tyr751) in DM1 vs CTL mice with severe muscle wasting. **Similar** in DM1 vs CTL mice with moderate muscle wasting.<br><br>Semi-quantitative total PDGFRβ levels:<br>Moderate muscle wasting: DM1 > CTL (p=ns)<br>Severe muscle wasting: DM1 > CTL ($p<0.01$)<br><br>Semi-quantitative p-PDGFRβ levels:<br>Moderate muscle wasting: DM1 < CTL (p=ns)<br>Severe muscle wasting: DM1 > CTL ($p<0.05$)<br><br>p-PDGFRβ/Total PDGFRβ ratio:<br>Moderate muscle wasting: DM1 < CTL (p=ns)<br>Severe muscle wasting: DM1 < CTL ($p<0.05$)
		RPPA analysis/Western blot	Stat3	Total Stat3 levels: **Increased** levels in DM1 vs CTL mice with severe muscle wasting. **Similar** levels in DM1 vs CTL mice with moderate muscle wasting.<br><br>p-Stat3 levels: **Increased** levels of p-Stat3 (Ser727) in DM1 vs CTL mice with severe muscle wasting. **Reduced** levels of p-Stat3 in proportion to total Stat3 levels in DM1 vs CTL mice with severe muscle wasting.<br><br>Semi-quantitative total Stat3 levels:<br>Moderate muscle wasting: DM1 ~ CTL (p=ns)<br>Severe muscle wasting: DM1 > CTL ($p<0.01$)<br><br>Semi-quantitative p-Stat3 levels:<br>Moderate muscle wasting: DM1 > CTL ($p<0.05$)<br>Severe muscle wasting: DM1 > CTL ($p<0.05$)<br><br>p-Stat3/Total Stat3 ratio:<br>Moderate muscle wasting: DM1 > CTL (p=ns)<br>Severe muscle wasting: DM1 < CTL ($p<0.05$)
	DM1: Human skeletal muscle tissue biopsies from patients with DM1: n=7	Western blot	AMPKα	Total AMPKα levels: **Similar** levels in DM1 vs CTL.<br><br>p-AMPKα levels: **Similar** levels of p-AMPKα (Thr172) in DM1 vs CTL

	CTL: Human skeletal muscle tissue biopsies from unaffected individuals; n=4			Semi-quantitative total AMPKα levels: DM1 < CTL (p=ns)<br>Semi-quantitative p-AMPKα levels: DM1> CTL (p=ns)<br><br>p-AMPKα /Total AMPKα ratio: DM1 > CTL (p=ns)
			PDGFRβ	Total PDGFRβ levels: **Increased** levels in DM1 vs CTL.<br><br>p-PDGFRβ levels: **Increased** levels of p-PDGFRβ (Tyr751) in DM1 vs CTL.<br><br>Semi-quantitative total PDGFRβ levels:DM1 > CTL ($p<0.001$)<br>Semi-quantitative p-PDGFRβ levels: DM1 > CTL ($p<0.05$)<br><br>p-PDGFRβ/Total PDGFRβ ratio: DM1 < CTL (p=ns)
			Stat3	Total Stat3 levels: **Similar** levels in DM1 vs CTL.<br><br>p-Stat3 levels: **Similar** levels of p-Stat3 (Ser727) in DM1 vs CTL<br><br>Semi-quantitative total Stat3 levels: DM1 > CTL (p=ns)<br>Semi-quantitative p-Stat3 levels: DM1 > CTL (p=ns)<br>p-Stat3/Total Stat3 ratio: DM1 < CTL (p=ns)
**Nakamori et al. 2017, Japan** [41]	DM1: Mice C2C12 myoblasts with 800 CUGexp RNA<br><br>CTL: Mice C2C12 myoblasts without CUGexp RNA	Western blot	IkBα	Total IkBα levels: **Decreased** levels in DM1 vs CTL.<br><br>p-IkBα levels: **Increased** levels of p-IkBα (Ser32) in DM1 vs CTL.<br><br>Semi-quantitative total IkBα levels: DM1 < CTL ($p<0.01$)<br>Semi-quantitative p-IkBα levels (% to CTL): DM1 > CTL ($p<0.01$)
		Western blot/ELISA	NF-kB	Total NF-kB levels: **Similar** in DM1 vs CTL.<br><br>p-NF-kB levels: **Increased** levels of p-NF-kB (Ser536) in DM1 vs CTL.<br><br>NF-kB activity: **Increased** nuclear localization and **increased** binding activity.<br><br>Semi-quantitative total NF-kB levels: DM1 > CTL (p=ns)<br><br>Semi-quantitative p-NF-kB levels: DM1 > CTL ($p<0.01$)<br><br>Semi-quantitative Nuclear NF-kB levels: DM1 > CTL ($p<0.01$)

				<u>Semi-quantitative NF-kB binding activity:</u> DM1 > CTL (<p<0.01)
		Western blot	PKR	<u>Total PKR levels:</u> **Similar** in DM1 vs CTL.<br><br><u>p-PKR levels:</u> **Increase** in p-PKR in DM1 vs CTL.<br><br><u>Semi-quantitative total PKR levels:</u> DM1 > CTL (p=ns)<br><br><u>Semi-quantitative p-PKR levels:</u> DM1 > CTL (p<0.01)
**Peric et al. 2014, Serbia** [42]	<u>jDM1:</u> Human CSF; n=27; Sex: 10F/17M; Age: 35±9.1 (Mean±SD); CTG Repeat Length: 859.2±311.8 (Mean±SD)<br><br><u>aDM1:</u> Human CSF; n=47; Age: 46.7±7.6 (Mean±SD); Sex: 27F/20M; CTG Repeat Length: 703.2±248.0 (Mean±SD)<br><br><u>CTL:</u> Human CSF; n=26; Age: 50.9±8.3 (Mean±SD); Sex: 16F/10M	Sandwich ELISA	Tau	<u>Total Tau levels:</u> Adult-onset with **similar** levels of Tau vs CTL and jDM1.<br><br><u>p-tau levels:</u> Adult-onset with **slight but not significant increase** in p-Tau (Thr181) vs CTL and jDM1.<br><br><u>Quantitative total Tau concentration (Mean±SD):</u><br>jDM1: 175.5±90.2 ng/l vs aDM1: 218.4±108.2 ng/l vs CTL: 178.9±38.6 ng/l (p=0.156)<br><br><u>Quantitative p-tau concentration (Mean±SD):</u><br>jDM1: 43.1±21.0 ng/l vs aDM1: 54.6±23.4 ng/l vs CTL: 47.8±23.7 ng/l (p=0.057)
**Ravel-Chapuis et al. 2017, Canada** [43]	<u>DM1:</u> HSA^{LR} mice EDL skeletal muscle tissue biopsies; n=4 (n for evaluation of activity=8) CTG repeat length: 250<br><br><u>CTL:</u> HSA^{SR} mice EDL skeletal muscle tissue biopsies	Western blot	Calcineurin	<u>Total Calcineurin levels</u>: **Increase** in ~2 fold in DM1 vs CTL (p<0.001)<br><br><u>Calcineurin activity:</u> **Increase** of ~2.5 fold in the phosphatase activity (p<0.001)
**Ravel-Chapuis et al. 2018, Canada** [44]	<u>DM1:</u> HSA^{LR} mice EDL skeletal muscle tissue biopsies; n range= 4-5; CTG repeat length: 250 n range= 4-5<br><br><u>CTL:</u> WT mice EDL skeletal muscle tissue biopsies; n range)= 4-5	Western blot	AMPK	<u>Total AMPK levels:</u> **Similar** in DM1 vs CTL (p=ns)<br><br><u>p-AMPK levels:</u> **Decrease** (~70%) in p-AMPK (Thr172) in DM1 vs CTL (p=0.02)<br><br><u>Semi-quantitative total AMPK levels:</u> DM1~CTL (p=ns)<br><br><u>p-AMPK/Total AMPK ratio:</u> DM1 < CTL (p=0.02)
	<u>DM1:</u> Human fibroblast cell line-derived myoblasts with DM1<br><br><u>CTL:</u> Human fibroblast cell line-derived myoblasts		AMPK	<u>Total AMPK levels:</u> **Similar** in DM1 vs CTL.<br><br><u>p-AMPK levels:</u> **Decrease** (~35%) in p-AMPK (Thr172) in DM1 vs CTL.<br><br><u>Semi-quantitative total AMPK levels:</u> DM1 > CTL (p=ns)

				<u>p-AMPK/Total AMPK ratio:</u> DM1 < CTL (p=0.02)
**Renna et al. 2017, Italy** [45]	<u>DM1:</u> Human patients' *biceps brachii* (BB) (n=4) and *tibialis anterior* (TA) (n=4) skeletal muscle tissue biopsies<br><br><u>CTL:</u> Healthy participants' BB (n=4) and TA (n=3) skeletal muscle tissue biopsies	Western blot	AKT	***Biceps brachii* (BB)**<br><u>Total AKT levels:</u> **Similar** levels in DM1 vs CTL.<br><u>p-AKT levels:</u> **Similar** levels of p-AKT (Thr308) in DM1 vs CTL.<br><br><u>Semi-quantitative total AKT levels:</u> DM1 > CTL (p=ns)<br><u>Semi-quantitative p-AKT levels:</u> DM1 > CTL (p=ns)<br><br>***Tibialis Anterior* (TA)**<br><u>Total AKT levels</u>: **Increased** in DM1 vs CTL.<br><u>p-AKT levels:</u> **Increased** values of p-AKT (Thr308) in DM1 vs CTL.<br><br><u>Semi-quantitative total AKT levels:</u> DM1 > CTL (p=0.02)<br><u>Semi-quantitative p-AKT levels:</u> DM1 > CTL (p=0.04)
			ERK1/2	***Biceps brachii* (BB)**<br><u>Total ERK1/2 levels</u>: **Increased** in DM1 vs CTL.<br><u>p-ERK1/2 levels:</u> **Similar** levels of p-ERK1/2 (Thr202/Tyr204) in DM1 vs CTL.<br><br><u>Semi-quantitative total ERK1/2 levels:</u> DM1 > CTL (p=0.02)<br><u>Semi-quantitative p-ERK1/2 levels:</u> DM1 > CTL (p=ns)<br><br>***Tibialis Anterior* (TA)**<br><u>Total ERK1/2 levels:</u> **Increased** in DM1 vs CTL.<br><u>p-ERK1/2 levels:</u> **Increased** levels of p-ERK1/2 (Thr202/Tyr204) in DM1 vs CTL<br><br><u>Semi-quantitative total ERK1/2 levels:</u> DM1 > CTL (p=0.01)<br><u>Semi-quantitative p-ERK1/2 levels:</u> DM1 > CTL (p=0.03)
			GSK3β	***Biceps brachii* (BB)**<br><u>Total GSK3β levels:</u> **Similar** values in DM1 vs CTL.<br><u>p-GSK3β levels:</u> **Similar** levels of p-GSK3β (Tyr216) in DM1 vs CTL.<br><br><u>Semi-quantitative total GSK3β levels:</u> DM1 > CTL (p=ns)<br><u>Semi-quantitative p-GSK3β levels:</u> DM1 > CTL (p=ns)<br><br>***Tibialis Anterior* (TA)**<br><u>Total GSK3β values</u>: **Decreased** in DM1 vs CTL.<br><u>p-GSK3β values:</u> **Increased** levels of p-GSK3β (Tyr216) in DM1 vs CTL.

				Semi-quantitative total GSK3β levels: DM1 < CTL (p=0.03)<br>Semi-quantitative p-GSK3β levels: DM1 > CTL (p=0.004)
			S6K1	***Biceps brachii* (BB)**<br>Total S6K1 levels: **Similar** values in DM1 vs CTL.<br>p-S6K1 levels: **Similar** levels of p-S6K1 (Thr421/Ser424) in DM1 vs CTL.<br><br>Semi-quantitative total S6K1 levels: DM1 < CTL (p=ns)<br>Semi-quantitative p-S6K1 levels: DM1 > CTL (p=ns)<br><br>***Tibialis Anterior* (TA)**<br>Total S6K1 levels: **Similar** levels in DM1 vs CTL<br>p-S6K1 levels: **Similar** levels values of p-S6K1 (Thr421/Ser424) in DM1 vs CTL<br><br>Semi-quantitative total S6K1 levels: DM1 > CTL (p=ns)<br>Semi-quantitative p-S6K1 levels: DM1 > CTL (p=ns)
	DM1: Human patients satellite cell-derived myotubes; n=5<br><br>CTL: Healthy participants satellite cell-derived myotubes; n=6		AKT	p-AKT levels: **Slower increase** in the activation of AKT (Thr308) upon insulin stimulation in DM1 vs CTL.<br><br>p-AKT/Total AKT ratio:<br>0 min.: DM1 ~ CTL (p=ns); 5 min.: DM1 < CTL (p=ns)<br>15 min.: DM1 < CTL (p=0.04); 30 min.: DM1 < CTL (p=ns)<br>Between DM1 vs CTL groups: p=0.04
			ERK 1	p-ERK 1 levels: **Similar** levels of p-ERK 1 (Thr202) upon insulin stimulation in DM1 vs CTL.<br><br>p-ERK 1/Total ERK 1 ratio:<br>0 min.: DM1 ~ CTL (p=ns); 5 min.: DM1 < CTL (p<0.05)<br>15 min.: DM1 < CTL (p=ns); 30 min.: DM1 < CTL (p=ns)<br>Between DM1 vs CTL groups: p=ns
			ERK 2	p-ERK 2 levels: **Decreased** levels of p-ERK 2 (Tyr204) upon insulin stimulation in DM1 vs CTL.<br><br>p-ERK 2/Total ERK 2 ratio:<br>0 min.: DM1 ~ CTL (p=ns); 5 min.: DM1 < CTL (p<0.05)<br>15 min.: DM1 < CTL (p=ns); 30 min.: DM1 < CTL (p=ns)<br>Between DM1 vs CTL groups: p=0.05

			GSK3β	p-GSK3β levels: **Not significant but increased** levels of p-GSK3β (Tyr216) upon insulin stimulation in DM1 vs CTL.<br><br>p-GSK3β/Total GSK3β ratio:<br>0 min.: DM1 ~ CTL (p=ns); 5 min.: DM1 > CTL (p=ns)<br>15 min.: DM1 > CTL (p=ns); 30 min.: DM1 > CTL (p=ns)<br>Between DM1 vs CTL groups: p=ns
			IRS1	p-IRS1 levels: **No increase** in p-IRS1 (Tyr612) upon insulin stimulation in DM1 vs **increase** in CTL. **Not significant but decreased** levels of p-IRS1 (Tyr896) in DM1 vs CTL.<br><br>p-IRS1/Total IRS1 (Tyr612) ratio:<br>0 min.: DM1 ~CTL (p=ns); 5 min.: DM1 < CTL (p=0.002)<br>15 min.: DM1 < CTL (p=0.01); 30 min.: DM1 ~CTL (p=ns)<br>Between DM1 vs CTL groups: p=0.01<br><br>p-IRS1/Total IRS1 (Tyr896) ratio:<br>0 min.: DM1 ~ CTL (p=ns); 5 min.: DM1 < CTL (p=0.04)<br>15 min.: DM1 < CTL (p=0.01); 30 min.: DM1 < CTL (p=ns)<br>Between DM1 vs CTL groups: p=ns
			S6K1	p-S6K1 levels: **Not significant but decreased** levels of p-S6K1 (Thr421/Ser424) upon insulin stimulation in DM1 vs CTL.<br><br>p-S6K1/Total S6K1 ratio:<br>0 min.: DM1 ~ CTL (p=ns); 5 min.: DM1 < CTL (p=ns)<br>15 min.: DM1 < CTL (p=ns); 30 min.: DM1 < CTL (p=ns)<br>Between DM1 vs CTL groups: p=ns
**Renna et al. 2019, Italy** [46]	DM1: Human patients' *biceps brachii* (BB) and *tibialis anterior* (TA) skeletal muscle tissue biopsies (n=8)<br><br>CTL: Healthy participants BB and TA skeletal muscle tissue biopsies (n=6)	Western blot	AKT	p-AKT levels: **Decreased activation** of AKT (Thr308/Ser473) upon insulin stimulation in DM1 vs CTL.<br><br>p-AKT fold change on basal level (Thr308): DM1 < CTL (p<0.01)<br><br>p-AKT fold change on basal level (Ser473): DM1 < CTL (p<0.01)
			AS160	p-AS160 levels: **Decreased** levels of p-AS160 (Thr642) upon insulin stimulation in DM1 vs CTL.<br><br>p-AS160 fold change on basal level: DM1 < CTL (p<0.01)
			ERK 1/2	p-ERK 1/2 levels: **Decreased** levels of p-ERK 1/2 (Thr202/Tyr204) upon insulin stimulation in DM1 vs CTL.

				p-ERK1/2 fold change on basal level: DM1 < CTL (p<0.01)
			FOXO1	p-FOXO1 levels: **Decreased** levels of p-FOXO1 (Thr24) upon insulin stimulation in DM1 vs CTL.<br><br>p-FOXO1 fold change on basal level: DM1 < CTL (p<0.01)
			IRS1	p-IRS1: **Decreased** levels of p-IRS1 (Tyr612) upon insulin stimulation in DM1 vs in CTL.<br><br>p-IRS1 fold change on basal level: DM1 < CTL (p<0.0001)
			mTOR	p-mTOR: **Decreased** levels of p-mTOR (Ser2448) upon insulin stimulation in DM1 vs CTL.<br><br>p-mTOR fold change on basal level: DM1 < CTL (p<0.01)
			S6K1	p-S6K1: **Decreased** levels of p-S6K1 (Thr421/Ser424) upon insulin stimulation in DM1 vs CTL<br><br>p-S6K1 fold change on basal level: DM1 < CTL (p<0.05)
**Rizzo et al. 2018, Italy** [47]	DM1: Human fetus-derived congenital DM1 myoblasts and myotubes; n=2; CDM13 CTG repeat length: 1800; CDM15 CTG repeat length: 3200<br><br>CTL: Human fetus-derived myoblasts and myotubes	Western blot	AKT	Total AKT levels: **Significantly decreased** levels in CDM15 vs CTL in day 0 of differentiation. **Similar** levels in CDM13 vs CTL in day 0 of differentiation.<br><br>Semi-quantitative total AKT levels: CDM13 < CTL (p=ns); CDM15 < CTL (p<0.05)
			S6K1	p-S6K1 levels: **Decreased** levels of p-S6K1 (Thr421/Ser424) at day 0 and day 3 of differentiation in DM1 (CDM15) vs CTL. However, there was an **increase** of p-S6K1 (Thr421/Ser424) at day 0 and day 3 of differentiation in DM1 (CDM13) vs CTL.<br><br>Semi-quantitative p-S6K1 levels:<br>d0: CDM15 < CDM13 > CTL; d3: CDM15 < CDM13 > CTL (p: NR)
**Sabater-arcis et al. 2020, Spain** [48]	DM1: Human fibroblast-derived myotubes with scrambled oligonucleotides; CTG repeat length: 1300<br><br>CTL: Human fibroblast-derived myotubes with scrambled oligonucleotides	Western blot	AKT	p-AKT levels: **Decreased** levels of p-AKT (Ser473) in DM1 vs CTL.<br><br>p-AKT/Total AKT ratio:<br>DM1: 0.910 ± 0.127 (Mean±SEM); CTL: 1.861 ± 0.140 (Mean±SEM) (p: NR)
			AMPK	p-AMPK levels: **Similar** levels of p-AMPK (Thr172) in DM1 and CTL.<br><br>p-AMPK/Total AMPK ratio:<br>DM1: 0.7906±0.1316 (Mean±SEM); CTL: 0.5272±0.1316 (Mean±SEM) (p: NR)

**Salisbury et al. 2008, USA** [49]	<u>DM1:</u> human patients' primary myoblasts and myotubes<br><br><u>CTL:</u> human primary myoblasts and myotubes	Western blot<br>EMSA assay	AKT	<u>Total AKT levels:</u> **Similar** levels in DM1 vs CTL (p: NR)<br><br><u>p-AKT levels:</u> **Increased** levels of p-AKT (Ser473) in the cytoplasm of DM1 vs CTL proliferating myoblasts. **Decreased** levels in DM1 vs CTL differentiating myotubes. (p: NR)<br><br><u>AKT activity:</u> **Increased** interaction with CUGBP1 in DM1 vs CTL myoblasts. (p: NR)
			CDK4	<u>Total CDK4 levels</u>: **Increased** levels in CTL differentiating myotubes, while levels remain **unchanged** in DM1 during differentiation. (p: NR)
			CUGBP1	<u>Total CUGBP1 levels:</u> **Decreased** levels in DM1 differentiating myotubes vs proliferating myoblasts. In CTL, CUGBP1 is **increased** in differentiating myotubes. (p: NR)<br><br><u>p-CUGBP1 levels:</u> **Increased** levels of p-CUGBP1 (Ser28) in DM1 vs CTL proliferating myoblasts. **Decreased levels** of p-CUGBP1 (Ser302) in DM1 vs CTL differentiating myotubes (p: NR)<br><br><u>CUGBP1 activity:</u> **Decreased** formation of CUGBP1–eIF2 complex during differentiation in DM1 vs CTL **Increased** interaction with Cyclin D1 mRNA in DM1 vs CTL myoblasts and myotubes. **Decreased** interaction with cyclin D3 in myotubes. (p: NR)
**Salvatori et al. 2005, Italy** [50]	<u>DM1:</u> Human patients skeletal muscle tissue biopsies; n=16<br><br><u>CTL:</u> Healthy participants skeletal muscle tissue biopsies; n=6	Immunohistochemistry<br>Western blot	DMPK	<u>Total DMPK levels:</u> **Decreased** levels in DM1 vs CTL.<br><br><u>Semi-quantitative total DMPK levels (fold change):</u><br>DM1: 1.96±0.24 (Mean±SEM) vs CTL: 3.62±0.69 (Mean±SEM) ($p=0.004$)
**Song et al. 2020, China** [51]	<u>DM1:</u> IPSC-derived satellite skeletal muscle cells (n=2) (DM1-03, DM1-13-3)<br><br><u>CTL:</u> IPSC-derived satellite skeletal muscle cells (n=1)	Western Blot	mTOR	<u>p-mTOR levels:</u> **Decreased** levels of p-mTOR (Ser2448) in DM1 vs CTL<br><br><u>p-mTOR/Total mTOR relative expression:</u> DM1-03 < DM1-13-3 < CTL: 1.0 ($p<0.05$)
	<u>DM1:</u> Human patients satellite skeletal muscle cells; n=3; Sex: 1M/2F; Age: 46±2 y (mean ± SD)<br><br><u>CTL:</u> Healthy participants satellite skeletal muscle cells; n=3; Sex: 2M/1F; Age: 48±3 years (mean ± SD)			<u>p-mTOR levels:</u> **Decreased** levels of p-mTOR (Ser2448) in DM1 vs CTL<br><br><u>p-mTOR/Total mTOR relative expression:</u> DM1 < CTL ($p<0.05$)
**Wang et al. 2009, USA** [52]	<u>DM1:</u> TAM+EpA960 RNA mice heart tissue	2D Gel/ Western Blot	CUGBP1	<u>Total CUGBP1 levels:</u> **Increased** in DM1 vs CTL. (p: NR)<br><br><u>p-CUGBP1 levels:</u> **Increased** phosphorylation levels of p-CUGBP1 in DM1. (p: NR)

	CTL: MCM mice heart tissue		PKCα/βII	p-PKCα/βII levels: **Increased** levels of p-PKC (Thr638/Thr641) in DM1 vs CTL. (p: NR)
**Wang et al. 2019, Taiwan** [53]	DM1: DMSXL mice brain tissue; CTG repeat length: >1000 CUG<br><br>CTL: WT mice brain tissue	Western blot	CUGBP1	p-CUGBP1 levels: **Decreased** levels of p-CUGBP1 (Ser302) in DM1 vs CTL. (p: NR)
	DM1: DMSXL adult mice diaphragm muscle tissue biopsies; CTG repeat length: >1000 CUGexp RNA<br><br>CTL: WT mice diaphragm muscle tissue biopsies		GSK3β	Total GSK3β levels: **Increased** in DM1 vs CTL. (p: NR)
	DM1: Human congenital DM1 myoblasts; n=3; CTG Repeat Length: ~2000<br><br>CTL: Healthy participants' myoblasts; n=3		GSK3β	Total GSK3β levels: **Increased** in DM1 vs CTL. (p: NR)
**González-Barriga et al. 2021, France** [54]	DM1: DMSXL mice primary oligodendrocytes<br><br>CTL: WT mice primary oligodendrocytes	Mass-spectrometry<br>2D Gel/ Western Blot	Capzb	p-CAPZB levels: **Increased** levels of p-CAPZB (Ser 263) in DM1 vs CTL (fold change: 2.778; p = 0.0089)
**Panaite et al. 2011, Switzerland** [55]	DM1: DMSXL mice spinal cord<br><br>CTL: WT mice primary spinal cord	Immunohistochemistry	Tau	Total Tau levels: **Decreased** in DM1 vs CTL. (p: NR)<br><br>p-Tau levels: **Increased** phosphorylation levels of p-Tau (Ser 396, Ser 404, Ser 199 and Ser 202) in DM1 vs CTL. (p: NR)
**Takahashi et al. 2001, Japan** [56]	DM1: COS-7 cells transiently expressing mutant DMPK $(CTG)_{130}$ cDNA<br><br>CTL: COS-7 cells transiently expressing WT DMPK $(CTG)_5$ cDNA	Western blot	DMPK	Total DMPK levels: **Decreased** levels in DM1 vs CTL in 20-30% ($p<0.05$).
**Fu et al. 1993, USA** [57]	DM1: Human adult DM1 patients skeletal muscle tissue biopsies; n=20<br><br>CTL: Human normal adult skeletal muscle tissue biopsies; n=7	Western Blot	DMPK	Total DMPK levels: **Decreased** levels in DM1 vs CTL. (p: NR)

**Furling et al. 2001, Canada** [58]	DM1: primary human DM1 skeletal muscle cell culture<br><br>CTL: primary human normal skeletal muscle cell culture	Western Blot	DMPK	Total DMPK levels: **Decreased** levels in DM1 vs CTL. (p: NR)
**Koga et al. 1994, Japan** [59]	DM1: Human adult DM1 patients skeletal muscle tissue biopsies; n=14<br><br>CTL: Human normal adult skeletal muscle tissue biopsies; n=NR	Western Blot	DMPK	Total DMPK levels: **Decreased** levels in DM1 vs CTL in about 36±36.9% (p: NR).
**Usuki et al. 2000, Japan** [60]	DM1: C2C12 cells stably expressing mutant DMPK $(CTG)_{160}$ cDNA<br><br>CTL: C2C12 cells stably expressing WT DMPK $(CTG)_5$ cDNA	Western Blot	c-Jun	Total c-Jun levels: **Similar** in DM1 vs CTL upon MeHg-induced cytotoxicity. (p: NR)<br><br>p-c-Jun levels: **Increased** phosphorylation levels of p-c-Jun (Ser 63) in DM1 vs CTL upon MeHg-induced cytotoxicity. (p: NR)
			Elk1	p-Elk1 levels: **Decreased** phosphorylation levels of p-Elk1 (Ser 383) in DM1 vs CTL upon MeHg-induced cytotoxicity. (p: NR)
**Timchenko et al. 2001, USA** [61]	DM1: human DM1 skeletal muscle cells<br><br>CTL: human normal skeletal muscle cells	Western Blot<br>Kinase assay	Cdk4	Total Cdk4 levels: **Similar** in DM1 vs CTL during myoblasts differentiation. (p: NR)<br><br>Cdk4 activity: **Increased** in DM1 vs CTL during myoblasts differentiation. (p: NR)
**Narang et al. 2000, Canada** [62]	DM1: Human adult DM1 patients skeletal muscle tissue biopsies; n=4<br><br>CTL: Human adult age-matched control skeletal muscle tissue biopsies; n=4	Western Blot	DMPK	Total DMPK levels: Significantly **Decreased** in DM1 vs CTL. ($p<0.005$)
	DM1: Human post-mortem congenital DM1 patients skeletal muscle tissue; n=4<br><br>CTL: Human post-mortem age-matched control skeletal muscle tissue; n=4			Total DMPK levels: **Similar** in DM1 vs CTL. (p: NR)
**Hernandez-Hernandez et**	DM1: PC12 cells stably expressing mutant DMPK $(CTG)_{190}$ cDNA	Western Blot	Tau	Total Tau levels: Slightly but significantly **Decreased** in DM1 vs CTL. ($p<0.05$)

**al. 2006, Mexico** [63]	CTL: PC12 cells stably expressing WT DMPK $(CTG)_5$ cDNA			p-Tau levels: Significantly **Increased** phosphorylation levels of p-Tau (Ser 396, Ser 404 and Thr231) in DM1 vs CTL. ($p<0.05$)

Abbreviatures: aDM1 – Adult phenotype; **AKT -** Protein kinase B; **AMPK** - 5' AMP-activated protein kinase; **AS160 -** TBC1 domain family member 4; **CaMKIIβM** - Ca^{2+}/calmodulin dependent protein kinase; **Capzb -** F-actin-capping protein subunit beta; **CDKN1B** - Cyclin-dependent kinase inhibitor 1B; **CDK4** – Cyclin-dependent kinase 4; **CDK6** - Cyclin-dependent kinase 6; **CHO-tTA** – chinese hamster ovary cells transfected with the TRE-GFP-CTG_{914} plasmid; **CSF** - Cerebrospinal fluid; **CTG** – Cytosine-Thymine-Guanine triplet; **CTL** – Control or Wild-type group; **CUGBP1** - CUG triplet repeat RNA binding protein 1; **CUGexp** – Expanded CUG; **DMPK -** Dystrophia myotonica protein kinase; **DMSXL -** Transgenic mice carrying >1,000 CTG repeats; **DM1**- Myotonic Dystrophy type 1 model group; **DM5 mice** - RNA toxicity model; **Dox** – Doxycycline; **dp-protein -** dephosphorylated protein; **d0** – Before exposure to differentiating medium; **d2/d3/d4/d6** – 2/3/4/6 days after exposure to differentiation medium; **EDL** - Extensor digitorum longus muscle; **eIF2α** - Eukaryotic translation initiation factor 2 subunit 1; **EpA960** – interrupted 960-CTG expansion model; **ERK 1/2** - Extracellular signal-regulated kinases 1/2; **Fed** – Mice food deprived for 12 hours followed by 4 hours of free access to food before sacrifice; **FOXO1** - Forkhead box protein O1; **GSK3** - Glycogen synthase kinase 3; **HSA^{LR}** - Transgenic mice expressing the human skeletal actin gene with long CUG repeat Length; **HSA^{SR}** - Transgenic mice expressing the human skeletal actin gene with short CUG repeat Length; **IGF-1** - Insulin-like growth factor 1; **IkBα** - Nuclear factor of kappa light polypeptide gene enhancer in B-cells inhibitor, alpha; **INPP4B** - Type II inositol 3,4-bisphosphate 4-phosphatase; **InsR** – Insulin Receptor; **IRS1** – Insulin Receptor Substrate 1; **jDM1** – Childhood/juvenile phenotype; **LBCLs -** Lymphoblastoid cell lines; **LKB1** - Liver kinase B1; **MCM** - MerCreMer (MCM) model; **MEK** - Mitogen-activated protein kinase kinase; **MeHg –** Methylmercury chloride**; mTOR** - Mammalian target of rapamycin; **n** – sample size; **n.d.** - not detected; **NF-kB** - Nuclear factor kappa-light-chain-enhancer of activated B cells; **PDGFRβ** - Platelet-derived growth factor receptor beta; **PERK** - Protein kinase R (PKR)-like endoplasmic reticulum kinase; **PKC** – Protein Kinase C; **PKM2** - Pyruvate kinase isozyme M2; **PKR** - Protein kinase R; **PLN** – Phospholamban; **p-protein -** phosphorylated protein; **PRKAR1B** - cAMP-dependent protein kinase type I-beta regulatory subunit; **p-state** - phosphorylation state; **PTPRF** - Receptor-type tyrosine-protein phosphatase F; **p38MAPK** - p38 Mitogen-activated protein kinase; **Rb** – Retinoblastoma protein; **RpS6** – Ribosomal protein S6; **SEM** - Standard error of the mean; **Stat3** - Signal transducer and activator of transcription 3; **S6K1** - Ribosomal protein S6 kinase beta-1; **TAK1** - Mitogen-activated protein kinase kinase kinase 7; **TAM** - Tamoxifen-inducible and heart-specific EpA960(R) RNA expression model; **TREDT960I -** Tetracycline-inducible transgene model; **TSC2** - Tuberous Sclerosis Complex 2; **ULK1** - Unc-51 like autophagy activating kinase; **WT** – Wildtype; **24hStarved/45hStarved** – Mice with 24/45 hours of food deprivation but free access to water before sacrifice.
